# Fundus hypopigmentation and choroidal thinning associated with tebentafusp therapy: report of a case and literature review

**DOI:** 10.1186/s12886-025-04274-7

**Published:** 2025-08-15

**Authors:** Jørgen Krohn, Liv Iren Hansen Vinnem, Ragnhild Wivestad Jansson, Oddbjørn Straume

**Affiliations:** 1 Department of Clinical Medicine, Section of Ophthalmology, University of Bergen, Bergen, Norway; 2 Department of Ophthalmology, Haukeland University Hospital, Bergen, Norway; 3 Cancer Clinic, Haukeland University Hospital, Bergen, Norway; 4 Department of Clinical Science, University of Bergen, Bergen, Norway

**Keywords:** Choroid, Choroidal thinning, Depigmentation, Hypopigmentation, Immune checkpoint inhibitors, Immunotherapy, Metastatic melanoma, Ocular adverse events, Tebentafusp, Uveal melanoma

## Abstract

**Purpose:**

To describe a novel case of progressive fundus hypopigmentation and choroidal thinning associated with tebentafusp monotherapy for metastatic uveal melanoma.

**Methods:**

Observational case report and review of the literature.

**Case presentation:**

A 69-year-old male was diagnosed with a choroidal melanoma, measuring 13.3 mm in diameter and 5.8 mm in thickness, in the left eye. Seven years after iodine-125 plaque brachytherapy, systemic imaging identified a solitary liver metastasis, which was laparoscopically resected. About one year later, two new liver metastases were detected. The patient was HLA-A*02:01 positive and started on tebentafusp. Except for transient fever, rash, and pruritus after the first cycles, the therapy was well tolerated. Fourteen months after initiation of tebentafusp, fundoscopy revealed marked hypopigmentation of both fundi and depigmentation of the regressed tumour in left eye. There were no signs of intraocular inflammation in either eye. Upon retrospective review of fundus photographs taken from baseline, the progressive fundus hypopigmentation and depigmentation of the tumour remnants first appeared after the initiation of immunotherapy. A corresponding evaluation of the optical coherence tomography scans of the previously untreated right eye revealed a significant reduction in central choroidal thickness over the same period. Full-field electroretinography demonstrated normal responses in the right eye and attenuated responses in the left eye. Screening for paraneoplastic antibodies was negative. During treatment, he also developed poliosis of the eyebrows and cilia, along with depigmented skin macules and patches. At the last visit, 11 years after the initial diagnosis and 26 months after starting tebentafusp, a repeat CT confirmed stable liver metastases with no new lesions. Both fundi appeared hypopigmented, and best corrected visual acuity was 1.0 in the right eye and hand movements in the left eye.

**Conclusions:**

Tebentafusp therapy can lead to diffuse fundus hypopigmentation and choroidal thinning, similar to what has been reported after immune checkpoint inhibition. The progressive choroidal hypopigmentation, without evidence of associated intraocular inflammation, indicates that glycoprotein 100, the target antigen of tebentafusp, is also expressed by normal choroidal melanocytes.

## Introduction

Over the last decade, immunotherapy has dramatically improved outcomes for patients with metastatic cutaneous melanoma and other advanced malignancies. Although both cutaneous and uveal melanoma are derived from melanocytes, their genetic, molecular, and clinical features are markedly distinct, which may explain their significantly different responses to immunotherapy [1]. The most commonly used immunotherapeutic agents are the monoclonal antibodies nivolumab and pembrolizumab, which target the programmed cell death 1 (PD-1) receptor on T cells and block its interaction with the programmed cell death ligand 1 (PD-L1), and ipilimumab, which targets the cytotoxic T-lymphocyte-associated protein 4 (CTLA-4) receptor [2, 3]. While these immune checkpoint inhibitors (ICIs) have shown some efficacy in metastatic uveal melanoma, particularly when used in combination to block both the PD-1 and CTLA-4 receptors, their therapeutic effectiveness remains limited compared to what has been observed in cutaneous melanoma [4, 5]. Tebentafusp is a novel T cell receptor bispecific fusion protein, designed for the treatment of HLA-A*02:01-positive patients with metastatic uveal melanoma. It activates T cells against the glycoprotein 100 (gp100) antigen presented by HLA-A*02:01 complexes on the surface of cells and has demonstrated survival rates superior to those reported with other treatments [4, 6, 7].

Both ICIs and tebentafusp stimulate T cells to attack tumour cells. However, normal cells expressing the same target molecules may also be affected, potentially inducing autoimmune responses in multiple organs [8]. Dermatologic adverse effects including maculopapular rash, pruritus, vitiligo-like depigmentation, and hypopigmentation of skin and hair, are frequently observed after initiation of both ICI and tebentafusp therapy [9, 10]. This indicates that the immunotherapy has also triggered an immune response against normal melanocytes, a phenomenon which has been associated with more favourable clinical outcomes [10–12].

Ocular adverse events associated with immune checkpoint inhibition include anterior uveitis, Vogt-Koyanagi-Harada (VKH)-like posterior uveitis, neuro-ophthalmic complications, as well as various orbital, ocular surface, and retinal manifestations [13, 14]. More rarely, fundus hypopigmentation without any signs of intraocular inflammation has been reported in patients receiving ICIs for metastatic melanoma [15]. Herein, we describe a patient who developed fundus hypopigmentation and choroidal thinning during tebentafusp therapy for metastatic uveal melanoma and provide a review of the literature on immunotherapy-related pigmentary fundus changes.

## Case presentation

A 69-year-old White male, retired welder, had experienced reduced vision and a visual field defect in his left eye over the past four months. His medical history included arterial hypertension and aortic valve stenosis. At initial examination, the patient's best corrected visual acuity (BCVA) was 1.0 in the right eye and 0.05 in the left eye, and the intraocular pressures were 14 mmHg and 15 mmHg, respectively. The anterior segment was unremarkable in both eyes. Fundoscopy of the right eye showed normal fundus pigmentation without any chorioretinal lesions. Posterior segment examination of the left eye revealed an elevated pigmented tumour in the lower temporal quadrant, with overlying orange pigment and minimal subretinal fluid, extending to the fovea. A shallow exudative retinal detachment was present in the inferior periphery. Fluorescein angiography showed normal retinal and choroidal vascular filling in the right eye, while the tumour in the left eye demonstrated patchy hyperfluorescence with late leakage. Ultrasound B-scan displayed a dome-shaped, acoustically hollow choroidal tumour measuring 13.3 mm in largest basal diameter and 5.8 mm in thickness, and A-scan demonstrated low to medium internal reflectivity, consistent with a choroidal melanoma. On magnetic resonance imaging (MRI) of the orbits, the lesion in the left eye was contrast-enhancing and appeared hyperintense on T1- and hypointense on T2-weighted images. MRI of the brain and computed tomography (CT) of the chest, abdomen, and pelvis revealed no evidence of systemic metastases. Genetic evaluation did not identify any known hereditary cancer syndromes.

The patient underwent iodine-125 plaque brachytherapy using an eccentrically placed COMS (Collaborative Ocular Melanoma Study) 16 mm plaque, with a prescribed dose of 90 Gy delivered to the tumour apex over 85 hours. Ophthalmic examinations were scheduled semi-annually for 5 years and annually for the next 5 years, while ultrasonographic screening for liver metastasis was planned 1-2 times per year for a total of 10 years. Follow-up examinations showed progressive regression of the choroidal melanoma and complete resolution of the exudative retinal detachment. At 9 months of follow-up, the tumour in the left eye was surrounded by choroidal atrophy, its thickness had decreased to 3.8 mm, and the BCVA was 0.2. Three and a half years after treatment, the tumour measured 2.3 mm in thickness and the BCVA was 0.05. Due to radiation-induced maculopathy, he started treatment with intravitreal bevacizumab every 4 to 6 weeks. Although the macular oedema resolved, his vision continued to deteriorate, and bevacizumab was discontinued after approximately one year.

At the 7-year follow-up, the tumour thickness was 1.7 mm and the visual acuity in the left eye was reduced to hand movements. Routine contrast-enhanced liver ultrasonography, followed by MRI, revealed a 7 mm subcapsular lesion in the right liver lobe. A percutaneous core needle liver biopsy identified melanin-containing cells, consistent with metastatic uveal melanoma. On positron emission tomography-computed tomography there were no other distant metastases, and the patient was scheduled for laparoscopic liver wedge resection. Due to worsening of the aortic valve stenosis, a transcatheter aortic valve implantation procedure was performed in advance to ensure a safe resection of the liver metastasis. Thereafter, the metastasis was resected, and the patient experienced an uneventful postoperative course. Histopathology revealed vacuolated melanoma cells and confirmed free surgical margins. One year after the aortic valve implantation, a mild paravalvular leak associated with haemolytic anaemia was managed with percutaneous plugging. At the same time, about nine years after initial diagnosis and one year after the liver wedge resection, contrast-enhanced liver CT and MRI revealed two small metastases, not previously seen, in the right lobe of the liver. A blood test showed that the patient was positive for the HLA-A*02:01 allele, and he was offered tebentafusp therapy as part of an early access program. Tebentafusp was then administered intravenously in weekly doses, according to the standard dosing regimen, starting with 20 mg, followed by 30 mg, and 68 mg thereafter. Shortly after each of the first three cycles, he experienced transient adverse effects, including fever, facial skin rash, and pruritus, which were treated symptomatically with antipyretics, antihistamines, and topical steroids; otherwise, the therapy was well tolerated. After two months on tebentafusp therapy, the patient reported episodes of sudden vision loss in his right eye, each lasting approximately 4-5 minutes, suggestive of amaurosis fugax. At that time, he was already on statins and anticoagulant therapy with apixaban. Physical examination was essentially normal, with no neurological deficits, carotid bruits, or significant findings on carotid ultrasound and echocardiography. MRI of the brain and CT angiography of the head and neck did not reveal any vascular abnormalities.

**Fig. 1:**
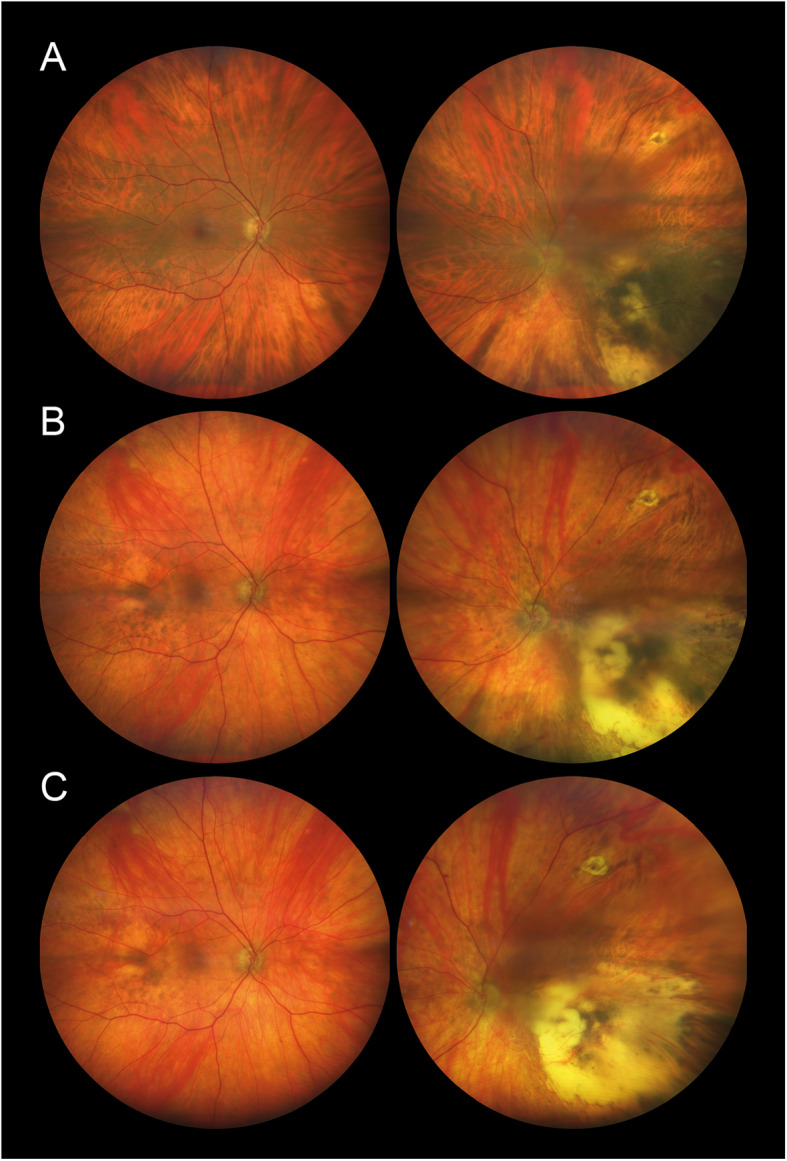
Wide-field fundus photographs of the patient, with the right eye displayed on the left and the left eye on the right. **A** Baseline imaging at the initiation of tebentafusp therapy, nine years after brachytherapy of the left eye. **B** Imaging at 14 months after tebentafusp initiation. **C** Imaging at 23 months after tebentafusp initiation. Note the progressive, diffuse hypopigmentation of both fundi and the increasing depigmentation of the regressed melanoma in the left eye

**Fig. 2:**
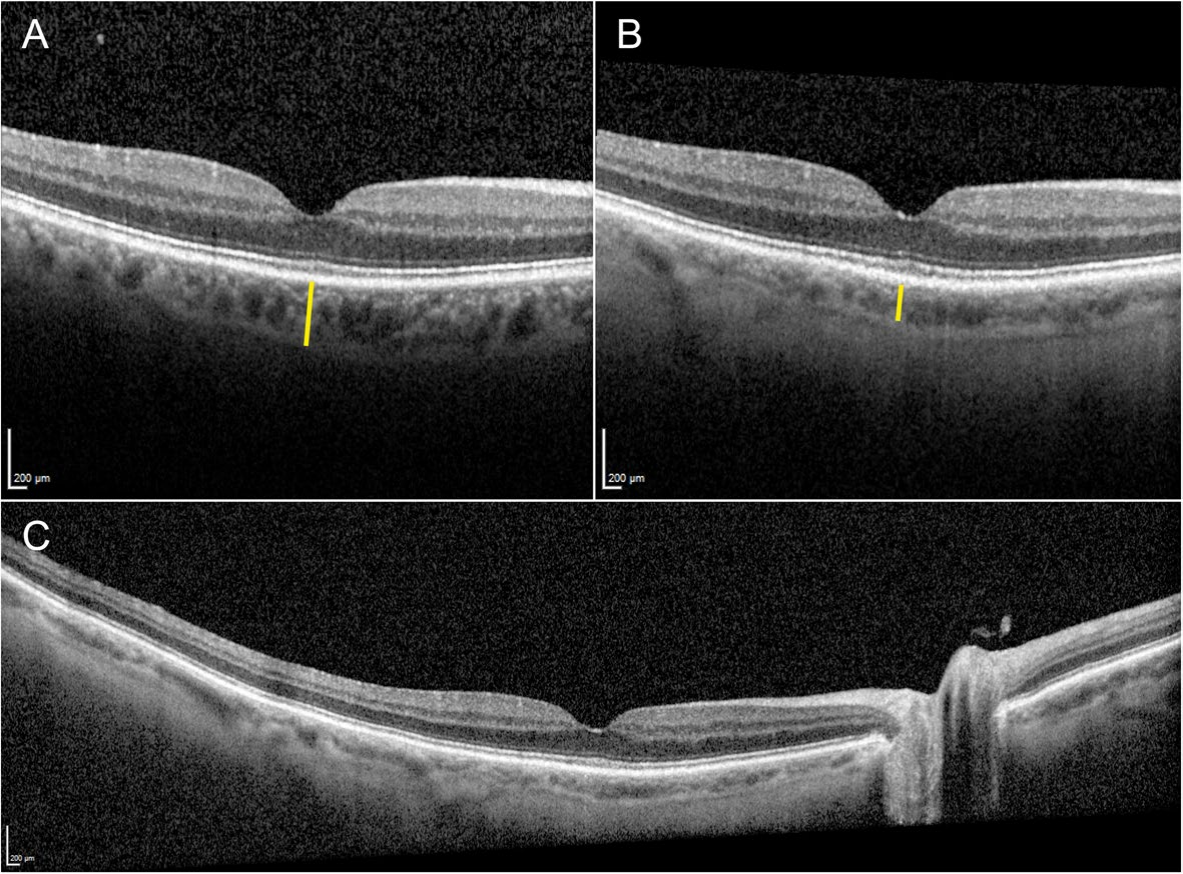
Optical coherence tomography scans of the right eye. The yellow vertical lines illustrate the change in central choroidal thickness. **A** Macular scan obtained four and a half years before the initiation of tebentafusp therapy. **B** Macular scan taken one year after starting tebentafusp therapy. **C** Wide-field scan from the same time point as (**B**), demonstrating generalised choroidal thinning

**Fig. 3:**
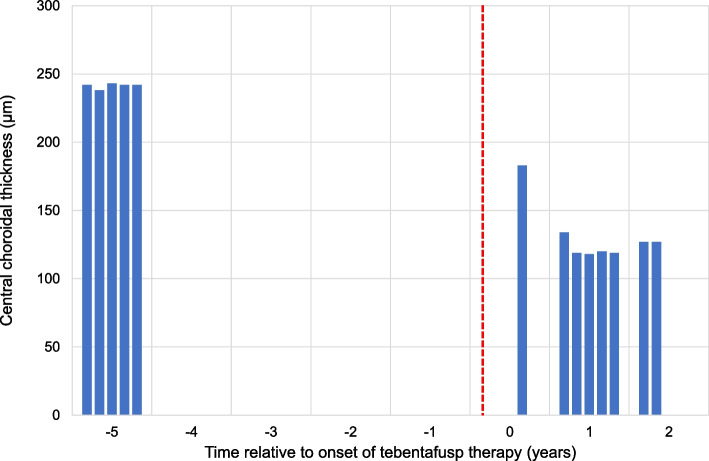
Bar chart showing the central choroidal thickness (μm) in the right eye at various time points relative to the onset of tebentafusp therapy. The vertical, dashed red line indicates the initiation of tebentafusp therapy

**Fig. 4:**
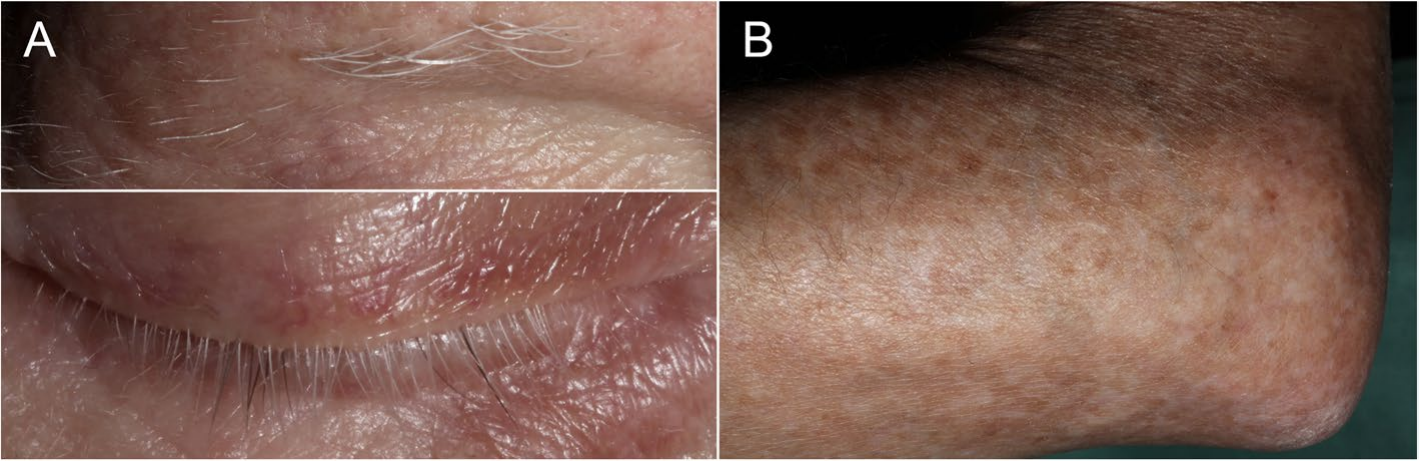
**A** Close-up photographs of the patient’s right eye region, taken two years after the initiation of tebentafusp therapy, demonstrating poliosis of the eyebrow and cilia. **B** Photograph of the patient’s left elbow, taken at the same time, showing irregular hypopigmented macules and patches

Ten years after the initial diagnosis and 14 months after the initiation of tebentafusp, his BCVA was 1.0 in the right eye and hand movements in the left eye. There were no signs of injection, anterior chamber cells, flare, or vitritis in either eye. Fundoscopy of the right eye revealed a markedly hypopigmented fundus with some irregular, faintly pigmented patches located temporal to the macula. Similarly, fundoscopy of the left eye showed general hypopigmentation of the fundus, with a depigmented, regressed choroidal melanoma in the inferotemporal region. Upon retrospective review of all fundus photographs routinely taken from baseline, the progressive pigment loss in both fundi and the tumour remnants first appeared after the initiation of tebentafusp therapy (Fig. 1). A corresponding evaluation of the optical coherence tomography (OCT) scans of the previously healthy, untreated right eye revealed a progressive reduction in central choroidal thickness, measured perpendicularly from Bruch’s membrane to the chorioscleral interface using the calliper tool on the Heidelberg Spectralis OCT (Heidelberg Engineering, Heidelberg, Germany) (Fig. 2). Five measurements taken during the fifth year prior to the initiation of tebentafusp, and seven measurements taken during the first two years after treatment, demonstrated a 49% reduction in mean central choroidal thickness, decreasing from 241 μm before to 123 μm after treatment (Fig. 3). Full-field flash electroretinography (ffERG), performed in accordance with the ISCEV (International Society for Clinical Electrophysiology of Vision) standard protocol, demonstrated normal photopic and scotopic responses in the right eye, whereas the irradiated left eye showed an expected reduction in the amplitudes and a delay in the peak times for both a- and b-waveforms. Over the course of treatment, he also developed poliosis of the eyebrows and cilia, along with skin changes characterised by circumscribed, depigmented macules and patches covering large areas of his body, particularly on the extremities (Fig. 4). Screening for paraneoplastic antibodies, including anti-recoverin, anti-HU, and anti-GAD65, was negative, and the patient’s serum vitamin A level was normal.

At the last visit, 11 years after the initial diagnosis and 26 months after starting tebentafusp, a repeat CT of the chest, abdomen, and pelvis confirmed stable liver metastases without any new lesions. At examination, the fundoscopic findings were essentially unchanged. Fluorescein and indocyanine green angiography of the right eye revealed normal retinal and choroidal circulation, the regressed tumour measured 1.3 mm in thickness, and the BCVA was 1.0 in the right eye and limited to hand movements in the left eye. Apart from clinical findings consistent with moderate heart failure, the patient was generally in good condition, reporting no new symptoms and doing well with ongoing treatment.

## Discussion

We present a novel case of progressive, diffuse fundus hypopigmentation and choroidal thinning associated with tebentafusp monotherapy for metastatic uveal melanoma. Except for the described fundus changes and dermatologic adverse effects, the treatment was well tolerated, and he has remained progression-free with stable liver metastases for more than two years while on tebentafusp therapy.

Ocular adverse events related to ICIs have been reported in approximately 1−3% of treated patients [14, 16]. These events are predominantly inflammatory in nature, with uveitis being the most common manifestation [17]. In a large pharmacovigilance analysis of ocular adverse events among patients treated with ICIs for various cancer types, uveitis emerged as the most reported condition, occurring in 15.1% of cases. Among these, non-specified uveitis accounted for 54.7%, anterior uveitis for 30.7%, and posterior uveitis for 10.5%. Additionally, ICI-associated VKH-like disease was identified in 8.3% of cases. The ocular adverse events, particularly uveitis, were most frequently observed in patients with melanoma and those receiving combination therapy with nivolumab and ipilimumab [18]. Clinical data on ocular adverse events associated with tebentafusp therapy are limited, and include dry eyes, pigmentary and inflammatory eyelid changes, and exudative subretinal fluid [10, 19].

Vogt-Koyanagi-Harada disease is characterised by bilateral granulomatous panuveitis and multifocal serous retinal detachments with choroidal thickening, often accompanied by systemic manifestations such as meningismus, tinnitus, alopecia, and vitiligo. Over time, patients typically develop granulomatous anterior uveitis and posterior segment depigmentation, known as “sunset glow fundus” [20]. In recent years, several reports have described VKH-like disease associated with immune checkpoint inhibition [18, 21–23], including cases leading to fundus depigmentation [24, 25]. However, progressive fundus hypopigmentation, without any signs of intraocular inflammation, is rare. Our literature review identified only seven patients with such pigmentary fundus changes related to cancer immunotherapy [15, 26–31]. A summary of these cases, including the present case, is provided in Table 1. All the patients were treated for metastatic melanoma, which is consistent with the literature on ocular adverse effects of ICI therapy and suggests an underlying difference in the risk of ocular complications between melanoma patients and those with non-melanoma cancers [14, 17, 22]. There was only one case of fundus hypopigmentation related to ICI therapy for metastatic uveal melanoma [31], which may be attributed to the lower prevalence and less frequent use of ICIs in uveal melanoma compared to cutaneous melanoma. Also, no conclusions can be made on whether the various ICIs carry different risks of fundus hypopigmentation, owing to variations in their availability and extent of use. However, four of the cases received combination therapy with ipilimumab and nivolumab, which is generally considered to pose a higher risk of ocular adverse effects compared to monotherapy [18, 32, 33]. As shown in Table 1, the pattern of fundus hypopigmentation differed among the ICI-treated patients. Four patients exhibited progressive diffuse fundus hypopigmentation [15, 28, 29, 31], of whom three also showed regression or disappearance of choroidal naevi [15, 28, 29]. The hypopigmentation was bilateral in all but two of these cases; one lacked information on the fellow eye [28], while the other had previously undergone enucleation [31]. Among the remaining patients, one displayed bilateral progressive fundus depigmentation in a petaloid pattern around the optic nerve [27]. Two patients presented with multiple circumscribed hypopigmented lesions; one with yellowish spots of varying sizes surrounding the optic nerve in the right eye and concurrent disappearance of choroidal naevi in both eyes [26], and the other with bilateral placoid hypopigmented lesions measuring 1–3 disc diameters [30]. In both patients, OCT revealed mild choroidal thickening corresponding to some of the hypopigmented lesions. Sarcoidosis and sarcoidosis-like reactions are known adverse events of immunotherapy that may also involve the eyes [14, 34]. The circumscribed lesions observed in the two patients may share similarities with sarcoid choroidal granulomas, but there was no evidence of sarcoidosis-related ocular inflammation. Interestingly, Ung & Gragoudas have reported a patient treated with nivolumab who presented with bilateral, creamy yellow choroidal lesions without ocular inflammation, and where systemic workup revealed pulmonary sarcoidosis, confirmed by biopsy [35]. The time from the start of immunotherapy to the detection of fundus hypopigmentation varied considerably among the cases in Table 1, ranging from six weeks to two years. However, since the hypopigmentation did not cause notable visual symptoms and was incidentally discovered during immunotherapy, no assumptions can be made about the duration from the start of treatment to the onset of pigmentary fundus changes. Several well-known ICI-related immunotoxicities were observed among the patients, including dermatologic findings such as vitiligo and poliosis affecting the eyebrows and cilia. These conditions ultimately developed in all patients and may result from shared autoimmune mechanisms targeting both melanoma cells and normal cutaneous melanocytes [36].

**Table 1:** Summary of reports on fundus hypopigmentation, without inflammatory manifestations, related to cancer immunotherapy

Authors, year, [Ref]	Primary cancer	Sex, age at diagnosis	Sites and time to detection of metastases	Immunotherapy agents	Patterns of fundus hypopigmentation	Time from start of immunotherapy to hypopigmentation	Visual symptoms	Other immunotoxicities	Follow-up, months	Visual acuity and systemic outcome
Sophie et al., 2019 [26]	Cutaneous melanoma	Male, 59	Axillary lymph nodes, 9 years	Nivolumab	Hypopigmented spots around the optic nerve OD; depigmentation of choroidal naevi OU	10 months	None	Elevated liver function tests, hypothyroidism, xerostomia, vitiligo	0	20/20 OU
Krohn et al., 2020 [15]	Cutaneous melanoma	Male, 61	Axillary lymph nodes, liver, 3 years	Nivolumab	Progressive, diffuse fundus hypopigmentation OU; regression of a choroidal naevus OS	11 months	None	Rash, pruritus, poliosis of eyebrows and cilia, skin and hair depigmentation	22	1.0 OU; regression of liver metastases, stable remission of axillary lymph nodes
Canestraro et al., 2020 [27]	Cutaneous melanoma	Female, 69	Brain, liver, lung	Ipilimumab, nivolumab, pembrolizumab	Progressive fundus depigmentation in a petaloid configuration around the optic nerve, involving the macula OU	NA	None	Progressive choroidal thinning OU; poliosis of cilia, vitiligo	26	20/25 OD, 20/30 OS
Foulsham et al., 2022 [28]	Cutaneous melanoma	Female, 64	NA	Ipilimumab, nivolumab	Progressive, diffuse fundus depigmentation and regression of a choroidal naevus OD	6 weeks	NA	Poliosis of cilia, widespread vitiligo	14	NA
Chatzichrlampous et al., 2022 [29]	Cutaneous melanoma	Male, 64	Lungs, 2 years	Nivolumab	Mild, diffuse fundus hypopigmentation OU; disappearance of a choroidal naevus OS	2 years	NA	Headaches, fatigue, poliosis of eyebrows and cilia, vitiligo	12	Regression of lung metastasis
Cotton et al., 2024 [30]	Cutaneous melanoma	Female, 47	Brain, abdomen, 4 years	Ipilimumab, nivolumab	Hypopigmented placoid lesions in the fundus OU	2 years	Floaters	Acute pancreatitis, autoimmune hypophysitis, poliosis of eyebrows and cilia	36	20/20 OD, 20/25 OS
Francis et al., 2024 [31]	Uveal melanoma	Male, 33	Liver, 6 years	Ipilimumab, nivolumab	Progressive, diffuse fundus depigmentation OS	NA	NA	Hepatitis, poliosis of cilia, vitiligo	27	Enucleated OD, 20/20 OS; partial metastasis response, progression-free
Current report	Uveal melanoma	Male, 69	Liver, 7 years	Tebentafusp	Progressive, diffuse fundus hypopigmentation OU, depigmentation of regressed choroidal melanoma OS	14 months	None	Progressive choroidal thinning OU; fever, rash, pruritus, poliosis of eyebrows and cilia, depigmented skin macules and patches	26	1.0 OD, hand movements OS; progression-free with stable metastases

*NA* not available, *OD* right eye, *OS* left eye, *OU* both eyes, *Ref* reference

The present case shows that tebentafusp therapy can lead to progressive, diffuse fundus hypopigmentation and choroidal thinning. These observations suggest that gp100 is expressed on normal choroidal melanocytes, consistent with immunohistochemical findings reported by de Vries et al. [37]. Except for radiation-related toxicities in the left eye, there were no signs of intraocular inflammation at any time point during treatment. A causal relationship between prior radiation to the left eye and the hypopigmentation in the right eye is considered highly unlikely, given the localised dose distribution inherent to brachytherapy and the delayed presentation of the fundus changes. The absence of focal fundus lesions and central nervous system symptoms makes prior sarcoidosis- or VKH-like disease unlikely. Exudative subretinal fluid associated with tebentafusp therapy has been recently reported [19], but this was not observed in our patient. Immunotherapy may increase the risk of developing paraneoplastic ocular syndromes such as carcinoma-associated retinopathy (CAR) and melanoma-associated retinopathy (MAR) [14, 38]. However, the normal ffERG, the absence of typical symptoms such as photopsia and nyctalopia, and the lack of outer retinal OCT abnormalities and paraneoplastic antibodies argue against ocular paraneoplasia in our patient. The diffuse fading of the characteristic choroidal striated or feathered pigmentation pattern suggests that the immune response primarily involves the choroid rather than the retinal pigment epithelium (RPE). This is further supported by the normal ffERG findings in the right eye and the absence of RPE abnormalities on OCT. Previous reports on fundus hypopigmentation associated with cutaneous vitiligo have also suggested that the pigment loss occurs in the choroid, as cutaneous and choroidal melanocytes share their embryological origin from the neural crest, while RPE cells are derived from the neuroectoderm [39, 40]. Additionally, the progressive choroidal thinning documented on OCT indicates that the choroid is the primary site of immune-mediated damage to pigmented cells. Given that central choroidal thickness is reported to decrease by 1.5 to 2 µm per year [41, 42], the observed reduction of 118 µm over approximately seven years in the present case cannot be explained by ageing alone. Choroidal thinning associated with immunotherapy has also been reported by Canestraro et al., who described a patient with progressive fundus hypopigmentation and choroidal thinning (leptochoroid) during immune checkpoint inhibition for metastatic cutaneous melanoma [27].

In summary, to our knowledge, this is the first reported case of fundus hypopigmentation and choroidal thinning associated with tebentafusp therapy. In view of the anecdotal nature of case reports and the inability to establish definitive causality, further reports and clinical experience are warranted to confirm this association. The literature review shows that similar fundus changes have also been observed following treatment with various ICIs. This indicates that gp100 (targeted by tebentafusp) and other melanocyte antigens (targeted by T cells whose PD-1 receptors are blocked by nivolumab or pembrolizumab) are expressed on normal choroidal melanocytes, and that CTLA-4 (targeted by ipilimumab) regulates immune responses within normal choroidal tissue. Because the fundus hypopigmentation and choroidal thinning cause few or no symptoms and are often detected incidentally, these adverse effects may be more common than previously considered. Since the fundus changes are typically bilateral and progress slowly, they can easily be overlooked without baseline or pre-treatment widefield fundus photographs and OCT scans for comparison. With the increasing use of immunotherapeutic agents, including tebentafusp, oncologists and ophthalmologists should be aware of these adverse effects to enhance our understanding of its underlying pathogenesis and clinical course.

## Data Availability

No datasets were generated or analysed during the current study.

## Abbreviations

BCVA: Best corrected visual acuity
CAR: Carcinoma-associated retinopathy
COMS: Collaborative Ocular Melanoma Study
CT: Computed tomography
CTLA-4: Cytotoxic T-lymphocyte-associated protein 4
ffERG: Full-field flash electroretinography
Gp100: Glycoprotein 100
HLA: Human leukocyte antigen
ICI: Immune checkpoint inhibitor
ISCEV: International Society for Clinical Electrophysiology of Vision
MAR: Melanoma-associated retinopathy
MRI: Magnetic resonance imaging
OCT: Optical coherence tomography
PD-1: Programmed cell death 1
PD-L1: Programmed cell death ligand 1
RPE: Retinal pigment epithelium
VKH: Vogt-Koyanagi-Harada
